# Dentistry at the Paris Exposition

**Published:** 1868-04

**Authors:** 


### SELECTED ARTICLES.

### ARTICLE VI.

### *Dentistry at the Paris Exposition.*

A QUARTER of a century ago, we became acquainted with the experiments of a dentist, in the effort to produce artificial teeth in a continuous gum, of a natural color and appearance, and which would not warp or become fissured in use. We sometimes visited his house and laboratory—almost daily—making minute inquiries and examinations of the materials, nature, and qualities, and progress of the work. During the last year or two we have not spoken to him, although living within a few minutes' walk of his residence, so neglectful are old friends of each other in this whirling, whirlpool city, unless stern business, or sterner illness, brings them together ; but we had heard of his sending specimens of his workmanship to the great Paris Exposition, and had been wondering in our own minds why we had not seen his name, with the grand cross of the legion of a dozen honors, we unexpectedly learned that the managers of the Exposition were not willing to place the efforts of genius, the work of professional men, with that of artificers. These labors continued for several years, through many discouragements and partial failures ; but, with a perseverance which belongs to men of true talent and high ability, he was able, at length, to arrive at a success which we felt confident would eventually be rewarded with a due appreciation, not only by the public at large, but by the dental profession of both hemispheres, which has been done. The jury on dentistry at the Champs de Mars have reported that "The specimens of 'Continuous gum sets of teeth, upon platinum plate,' by John Allen & Son, of New-York, are incomparably the most beautiful pieces exhibited." This report has been corroborated by various newspapers correspondents writing from Paris.

The New-York *Times*, of May 14th, 1867, has an article from its correspondent, saying of American dentistry, "It is beyond all competition. The display of artificial teeth, the perfect imitation of nature, in gums, and palate, or roof, are wonderful. But the French are equally great in eyes and ears."—*Journal of Health.*

---

